# Site-specific temporal variation of population dynamics in subalpine endemic plant species

**DOI:** 10.1038/s41598-022-23903-5

**Published:** 2022-11-10

**Authors:** Hyungsoon Jeong, Yong-Chan Cho, Eunsuk Kim

**Affiliations:** 1 School of Earth Sciences and Environmental Engineering, Gwangju Institute of Science and Technology, Gwangju, 61005 Korea; 2 Conservation Center for Gwangneung Forest, Korea National Arboretum, Pocheon, 11186 Korea

**Keywords:** Conservation biology, Population dynamics

## Abstract

Endemic plants in high mountains are projected to be at high risk because of climate change. Temporal demographic variation is a major factor affecting population viability because plants often occur in small, isolated populations. Because isolated populations tend to exhibit genetic differentiation, analyzing temporal demographic variation in multiple populations is required for the management of high mountain endemic species. We examined the population dynamics of an endemic plant species, *Primula farinosa* subsp. *modesta*, in four subalpine sites over six years. Stage-based transition matrices were constructed, and temporal variation in the projected population growth rate (λ) was analyzed using life table response experiments (LTREs). The variation in λ was primarily explained by the site × year interaction rather than the main effects of the site and year. The testing sites exhibited inconsistent patterns in the LTRE contributions of the vital rates to the temporal deviation of λ. However, within sites, growth or stasis had significant negative correlations with temporal λ deviation. Negative correlations among the contributions of vital rates were also detected within the two testing sites, and the removal of the correlations alleviated temporal fluctuations in λ. The response of vital rates to yearly environmental fluctuations reduced the temporal variation of λ. Such effects manifested especially at two sites where plants exhibited higher plasticity than plants at other sites. Site-specific temporal variation implies that populations of high mountain species likely exhibit asynchronous temporal changes, and multiple sites need to be evaluated for their conservation.

## Introduction

High mountain habitats are at the edge of climate gradients and are decreasing rapidly due to climate change^1,2^. Plant populations near the summit are projected to be at a high risk of extinction because the area with suitable environmental conditions is shrinking, and consequently, the opportunities for environmental tracking via migration are decreasing^3^. Notably, the high mountain habitat is a hotspot of endemic plant species^4,5^. Because endemic species are a critical component of biodiversity in high mountain habitats, characterizing the population dynamics of high mountain endemic species is important for biodiversity conservation.

High mountain species often occur in small, isolated populations, which makes them vulnerable to extinction like endemic species with a narrow geographic range^6,7^. A major factor influencing the extinction risk of such populations is the temporal variation in abundance due to environmental stochasticity^8–10^. Characterizing the responses of vital rates (survival, growth, and reproduction) to temporally fluctuating environments and the effects of their variability on population growth rates (λ) can provide information on the population viability of high mountain plant populations^11^.

When population demography is examined across a species’ geographic range, the contributions of vital rates to λ tend to differ among populations. In addition, the contributions of vital rates to λ often exhibit negative covariation across populations along environmental gradients, a phenomenon called demographic compensation^12–14^. Similarly, vital rates are predicted to co-vary in response to temporal environmental changes^12,15^. Temporal demographic compensation is expected to buffer temporal λ variation, but whether high mountain plant populations would exhibit temporal demographic compensation remains unknown.

The plastic responses of vital rates to temporal environmental fluctuations determine temporal demographic variation^16^. Geographically isolated populations, including high mountain plant populations, are often genetically differentiated because of limited gene flow or adaptation to their habitats^17,18^. Consequently, vital rates of high mountain populations might exhibit differential plastic responses to environmental fluctuations, which results in distinctive temporal variations in population demography^19–21^. Assessing temporal demographic variation in multiple populations is necessary for the conservation management of high mountain species.

*Primula farinosa* subsp. *modesta*, a perennial polycarpic herb, is a subalpine plant endemic to South Korea. *P. farinosa* is native to Northern Europe and Northern Asia^22^ and produces heterostylous flowers with thrum-eyed or pin-eyed flowers, with incompatibility within the same flower morphs^23^. The habitat of *P. farinosa* on the Korean Peninsula is grasslands or crevices of rocks higher than 1000 m above sea level. Similar to alpine endemic plant species in the Alps^24^, *P. farinosa* subsp. *modesta* is suggested to inhabit the entire Korean Peninsula in the early Pleistocene, but their distribution has shrunk into subalpine habitats because of increasing temperatures^25^. Gene flow among populations on different mountaintops is highly limited owing to long-term geographic isolation, which results in relatively high genetic differentiation among Korean populations^25^. When plants are grown in growth chamber environments, natural populations exhibit differential growth and survival in response to temperature and nitrogen deposition^20^.

**Figure 1:**
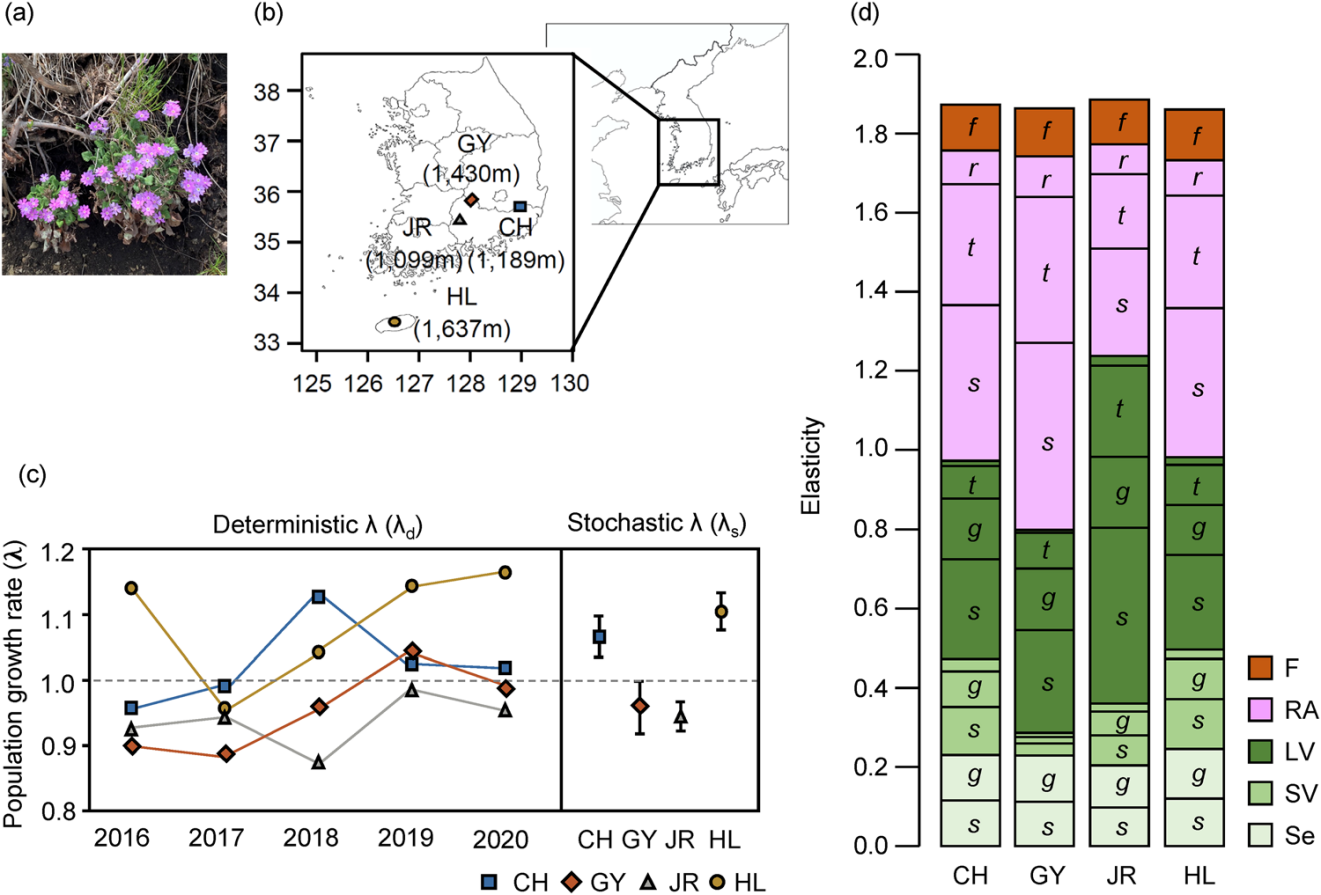
A photograph of *Primula farinose* (**a**), a map of study populations (**b**), projected population growth rate (λ) (**c**), and elasticity values (**d**). (**c**) Deterministic λ of each site and year (left side) and stochastic λ with 95% confidence intervals for each site (right side). (**d**) Vital rates elasticity values of the mean matrix across years for each site. Life stages are divided into seedling (Se), small vegetative plants (SV), large vegetative plants (LV), reproductive adults (RV), and fecundity (F). *s* survival, *g* growth, *r* retrogression, *t* stasis, *f* seedling emergence.

This study conducted a demographic survey from 2016 to 2021 and analyzed the temporal demographic variation of *P. farinosa* populations on four distant mountaintops in Korea (Fig. 1a,b, Supplementary Table S1). The life cycle of *P. farinosa* was divided into four life stages, including seedling (Se), small vegetative plant (SV), large vegetative plant (LV), and reproductive adult (RA) following Lindborg and Ehrlén^26^. Stage-structured transition matrices were constructed for each site and year^27,28^ (Supplementary Tables S2, S3). Following Villellas et al.^12^ and Jongejans et al.^29^, the elements of the transition matrices were decomposed into five vital rates, including survival (*s*_*i*_), growth conditional on survival (*g*_*ij*_), retrogression conditional on survival and not growth (*r*_*ij*_), stasis in the same life stage conditional on survival and not growth (*t*_*ij*_), and seedling emergence (*f*). We employed a life table response experiment (LTRE) to decompose the variation of λ into site and year effects and further decomposed into the LTRE effects of vital rates^29,30^. To evaluate whether the LTRE effects of vital rates stimulate or buffer the temporal variation of λ, we adopted two methods. First, we assessed the correlation between the LTRE effect of the vital rates and the temporal variation of λ. A negative correlation indicates that the variation in these vital rates buffers the annual variation in λ^8,30^. Second, the effects of demographic compensation on the temporal variation of λ were examined by evaluating the negative correlations between the LTRE effects of vital rates^12^.

Specifically, the following questions were addressed: (1) How do vital rates contribute to the yearly variation of λ? (2) Do the LTRE effects of vital rates have a negative correlation with the yearly deviation of λ? (3) Do the LTRE effects of vital rates have negative correlations with each other? (4) Do the correlation patterns differ among study sites?

## Results

### Population growth rate and elasticity of vital rates

The deterministic population growth rate (λ_d_) fluctuated from 2016 to 2021, and no consistent tendency in yearly variation was detected (Fig. 1c). Stochastic growth rates (λ_s_) over six years differed among the four sites (Fig. 1c). The λ_s_ in the CH (λ_s_ = 1.065) and HL (λ_s_ = 1.106) sites were greater than one, but those in the GY (λ_s_ = 0.960) and JR (λ_s_ = 0.948) sites were lower than one, indicating that the population size decreased. The vital rate elasticities of the LV and RA stages were higher than those of the Se and SV stages (Fig. 1d).

### Life-table response experiment

**Figure 2:**
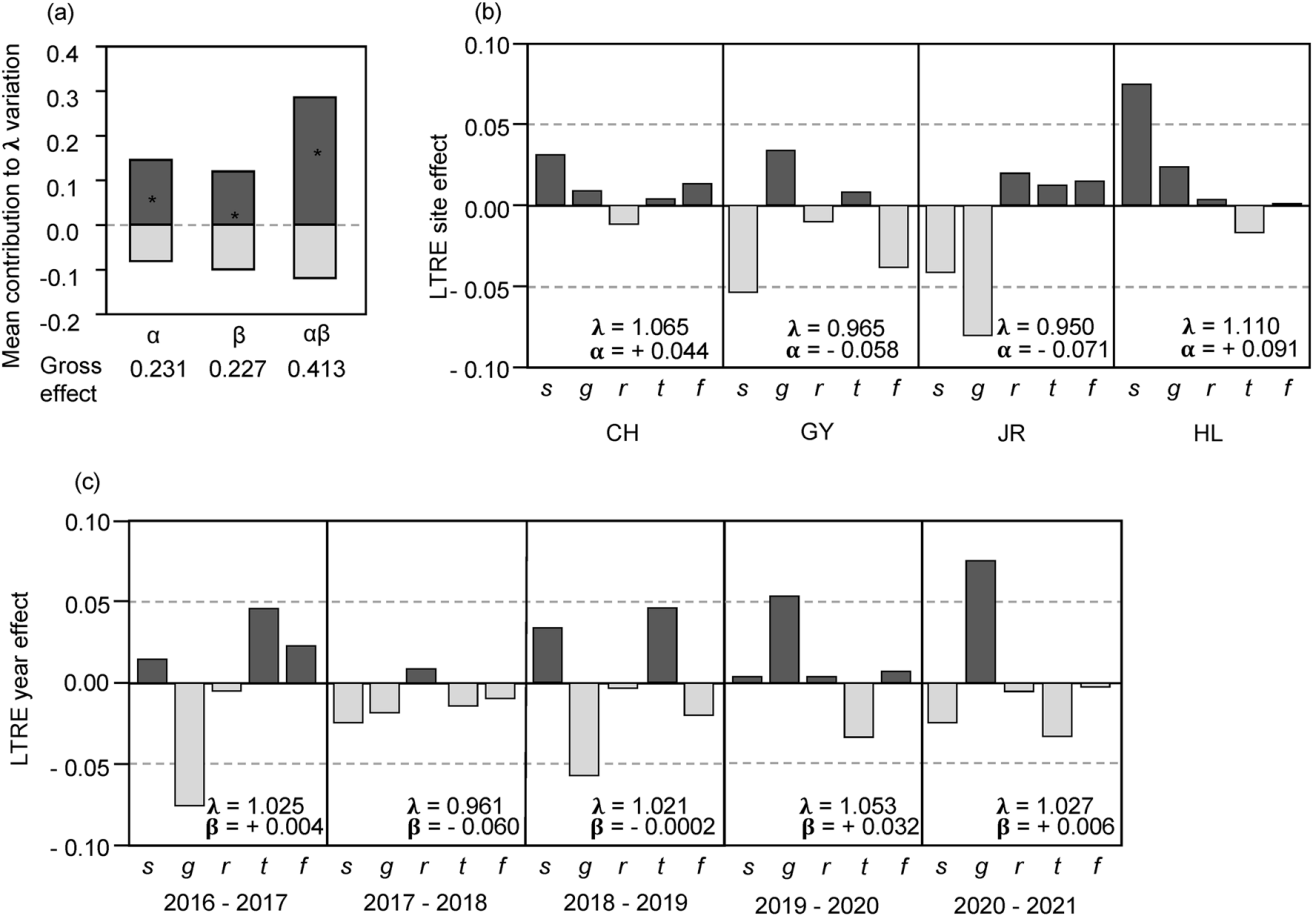
Results of life-table response experiment (LTRE) analysis. (**a**) Mean contribution of site (α), year (β), and their interaction (αβ) on the variation in population growth rate (λ). Net effects are denoted by an asterisk (*). Dark grey bars represent contributions with the same direction as the net effects; grey bars are those with the opposite direction. (**b**) Contribution of vital rates to the LTRE site effect. (**c**) Contribution of vital rates to the LTRE year effect. *s* survival, *g* growth, *r* retrogression, *t* stasis, *f* seedling emergence.

Two-way decomposition (site and year) of the variation in λ revealed similar gross and net effects of site and year (Fig. 2a). The effect of the site × year interaction was larger than the site and year main effects, indicating differential temporal variation in population dynamics among sites (Fig. 2a).

When the site effect on λ variation was decomposed into five vital rates, the contribution of survival and growth was the largest among the vital rates (Fig. 2b). The negative effect of survival induced a low λ at sites JR and GY. Growth also negatively affected λ at site JR, resulting in the lowest λ among the four sites. Relatively little effect was detected for stasis and seedling recruitment. In contrast, the growth showed the greatest contribution to the yearly λ variation (Fig. 2c). Except in 2017, the effect of stasis was in the opposite direction to that of growth, suggesting that it likely buffered the impact of growth. Survival and seedling recruitment also exhibited opposite effects on growth in 3 of the 5 years. In 2017 and 2019, four of the five vital rates contributed to the temporal variation in the same direction, resulting in the greatest positive or negative year effects.

**Figure 3:**
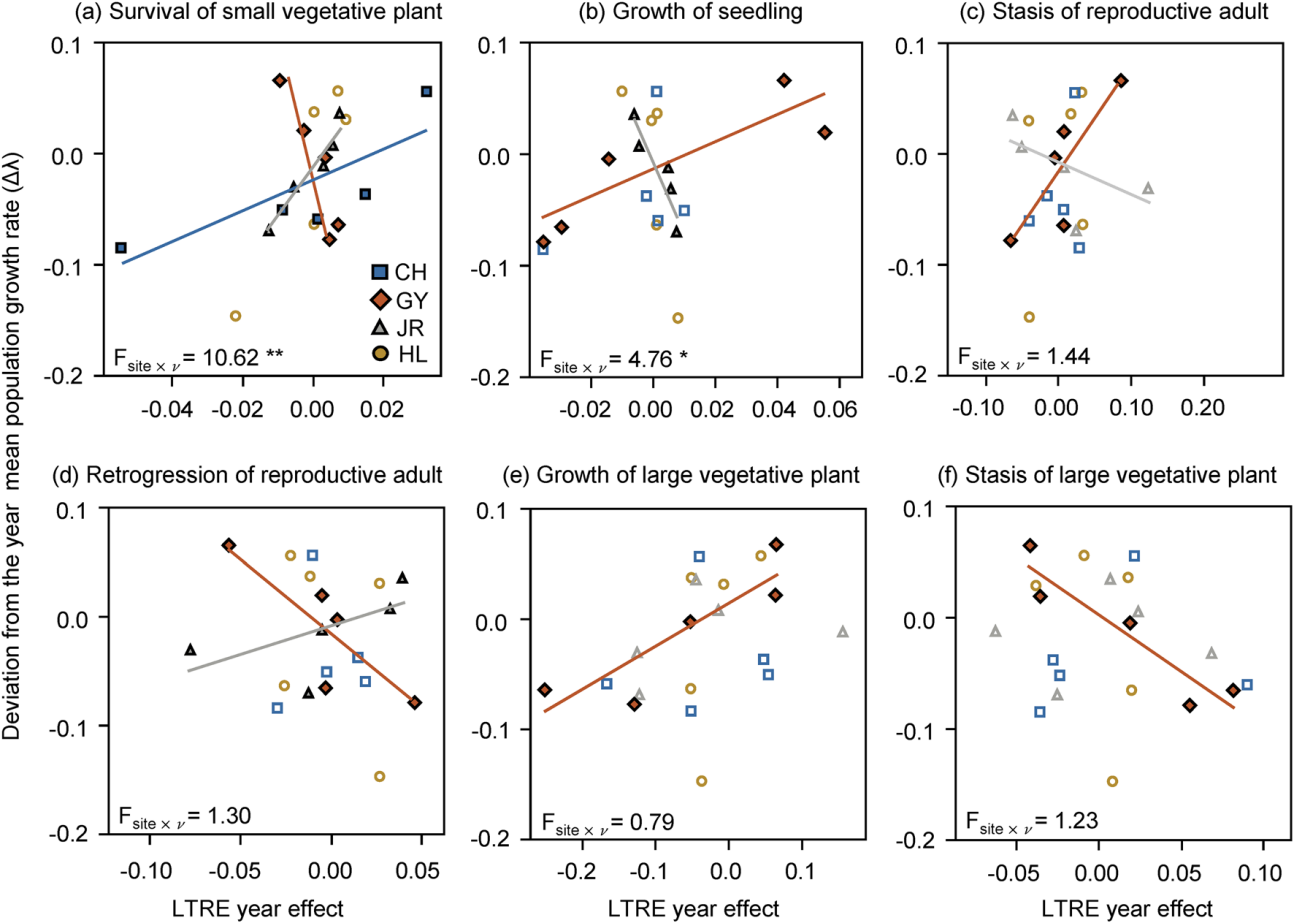
Correlation diagrams between variation in population growth rate (λ) and variation in the vital rate of LTRE contribution to year effect. Separate analyses were conducted for each life stage. Spearman’s correlation coefficients were calculated for each site and correlations with statistical significance (*P* < 0.05) by permutation test are represented by solid symbols and solid trend lines. Results of analyses of covariance are also provided. *P < 0.05, **P < 0.01.

Sites exhibited distinctive correlations between the LTRE effects of vital rates and the yearly deviation of λ (Fig. 3). The LTRE effect of SV survival on the population growth rate had a positive correlation with yearly λ deviation at sites CH and JR but a negative correlation at site GY (Fig. 3a). Thus, the increasing contribution of SV survival reduced the yearly λ deviation at site GY but magnified the yearly variation at sites CH and JR. An opposite pattern was found for the contribution of Se growth and RA stasis, which positively correlated with yearly λ deviation at site GY but negatively correlated with it at site JR, although the vital rate by site interaction was not statistically significant for RA stasis (Fig. 3b,c). The LTRE effect of RA retrogression showed a similar pattern to the effect of SV survival (Fig. 3d). The yearly deviation of λ at the GY sites showed a significant negative correlation with LV stasis and a positive correlation with LV growth, although other sites did not (Fig. 3e,f). No significant correlations were detected for other vital rates (Supplementary Fig. S1).

### Demographic compensation

**Figure 4:**
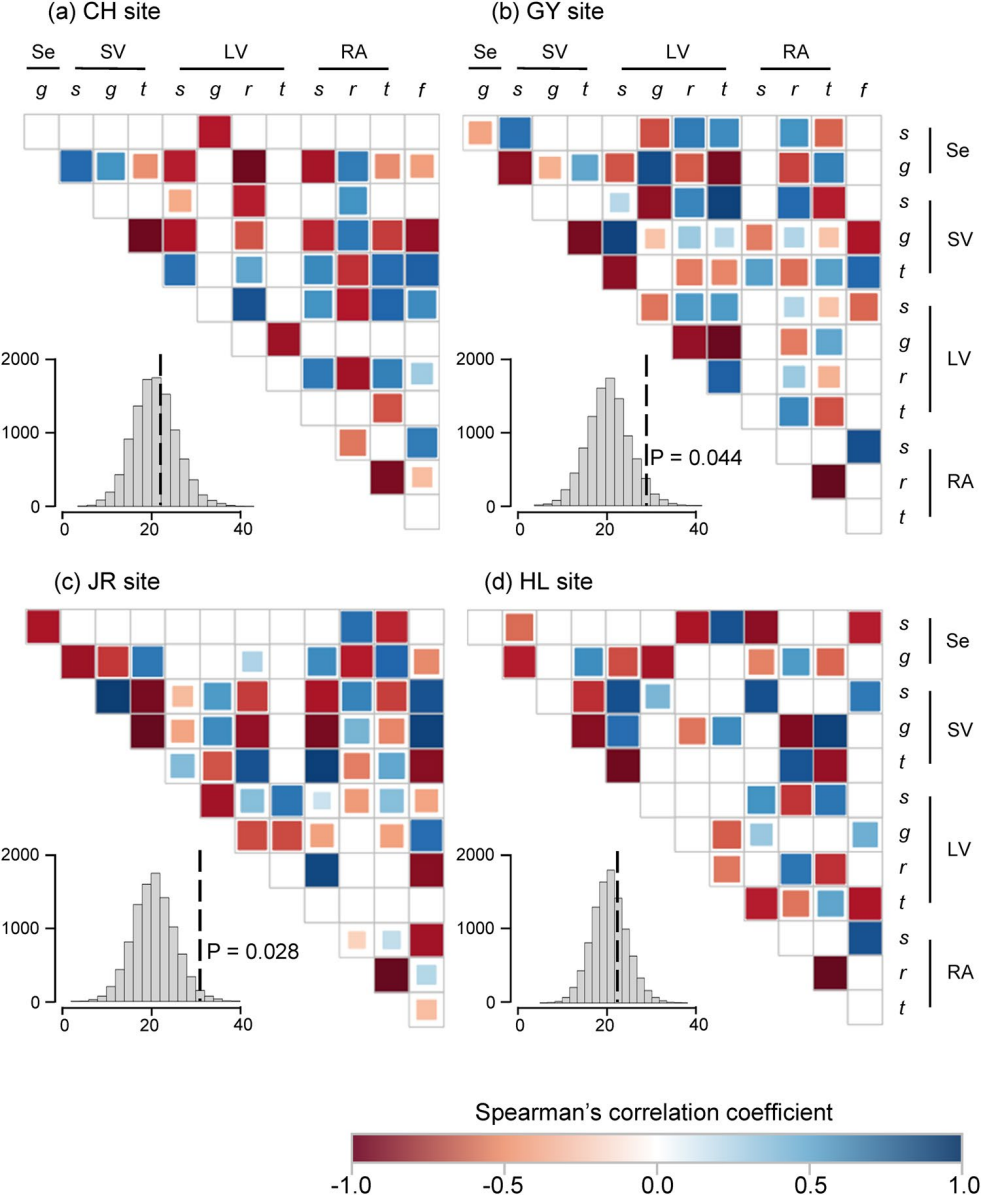
Correlogram of Spearman’s correlation coefficients between net contributions of different vital rates for each site. Negative correlations are in red and positive correlations are in blue. Colored boxes indicate significant correlations at *P* < 0.05. The histograms in the insets represent the distribution of the number of negative correlations expected by chance, and dotted line represents the observed number of significant negative correlations. *s* survival, *g* growth, *r* retrogression, *t* stasis, *f* seedling emergence.

Significant negative temporal correlations were detected between the LTRE effects of vital rates at each site (Fig. 4). The number of negative correlations (P < 0.05) were as follows: 23 at site CH, 29 at site GY, 31 at site JR, and 23 at site HL. However, the number of negative correlations was higher than expected by chance only at the JR (*P* < 0.001) and GY (*P* < 0.05) sites (Fig. 4). Two negative correlations, that is, correlations between growth and stasis within the SV and LV life stages, were detected at all sites. At the GY and JR sites, vital rates in distinctive life stages were negatively correlated (Fig. 4b,c), and most of them were between growth and survival or stasis. The growth of the Se stage was negatively correlated with the survival and growth of the SV stage; the survival of the Se stage was correlated with stasis of the RA stage. In addition, SV growth was negatively correlated with RA survival and stasis, and SV survival was negatively correlated with RA stasis.

**Figure 5:**
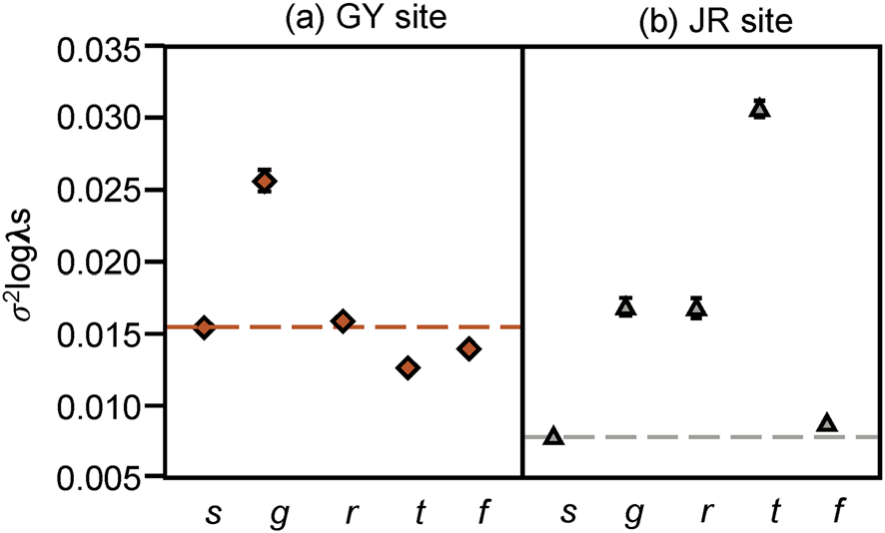
Variation of stochastic population growth rate (σ^2^_logλs_) obtained by permuting each vital rate. Mean value and 95% confidence intervals with 1000 permutations are provided. The horizontal dashed line indicates observed σ^2^logλs. Mean σ^2^_logλs_ higher than the observed value indicates that the corresponding vital rate effectively reduces σ^2^_logλs_ through demographic compensation. *s* survival, *g* growth, *r* retrogression, *t* stasis, *f* seedling emergence.

The negative correlations that buffered the yearly λ variation also differed between the GY and JR sites. When negative correlations related to the growth were permuted, the variance of stochastic λ (σ^2^_logλs_) increased at site GY, indicating that it buffered the yearly λ variation there (Fig. 5a). At site JR, negative correlations related with stasis and retrogression were also involved in alleviating the yearly λ variation (Fig. 5b).

## Discussion

In this study, we analyzed the temporal variation in λ in a subalpine endemic plant species, *P. farinosa*. Growth and survival contributed to the yearly λ variation, but the direction of their contributions was opposite. Some LTRE effects of vital rates have a negative correlation with yearly λ deviation, and they have negative correlations with each other over time. These results suggest that the temporal variation of some vital rates can reduce temporal λ variation. Vital rates reducing temporal λ variation differed among the testing sites, indicating that differential demographic mechanisms seem to shape temporal λ variation.

### Contribution of vital rates to temporal variation of λ

The analysis of the LTREs revealed that the year effect on the variation of λ was comparable to the site effect (Fig. 2), which is consistent with a study showing that the temporal variation was as significant as the spatial variation in 38 perennial plant species^10^. Although significant temporal variations tend to be detected when populations at a close distance of less than a few kilometers are examined, our testing populations separated by more than 56 km show a similar pattern^10,31^. Plant life history is another attribute affecting temporal demographic fluctuations, such that non-clonal herbaceous plants such as *P. farinosa* tend to exhibit high temporal variation^29,32^.

Growth was a critical vital rate contributing to the yearly variation in λ (Fig. 2c) even though growth elasticity was smaller than survival and stasis elasticities (Fig. 1d). The survival of post-seedling plants has been suggested as a vital rate affecting temporal λ variation in long-lived perennials^8^. However, for short-lived perennials, growth could be another vital rate that increases temporal variation^29,32^.

Notably, the directions of the survival and stasis effects were opposite to the growth effect in 4 out of 5 years (Fig. 2c), which can buffer the growth effects on the yearly λ variation. In addition, several negative correlations over 6 years were detected between growth and survival or between growth and stasis (Fig. 4). When plants from testing *P. farinosa* populations were grown in a growth chamber environment, increased temperature stimulated plant growth but decreased survival rates^20^. Similar trends have been reported for several alpine plant species^33–35^. Although diverse environmental factors fluctuate over the years, changing temperature would be a factor inducing opposite responses in survival and growth and, consequently, their opposite contributions to the temporal variation of λ.

### Site-specific temporal demographic variation

In the LTRE results, the site-by-year interaction was higher than the main effect of site and year (Fig. 2), indicating that the population growth rate fluctuated asynchronously among sites^29,30^. According to Dibner et al.^8^, the degree of temporal λ variation depends on diverse ecological factors, including negative density dependence^36^, being asynchronous within-population responses to environmental fluctuations^11^, and fine-scale source-sink dynamics^37^. In addition to these factors, a vital rate at which the LTRE effect is negatively correlated with temporal λ deviation is suggested to buffer temporal λ variation^30,32^. In this study, the testing sites exhibited differential correlations (Fig. 3) such that statistically significant negative correlations were only detected at the GY and JR sites but not in the CH and HL sites. Another demographic factor affecting temporal λ variation is demographic compensation, that is, the opposing responses of vital rates to environmental fluctuations^12^. Demographic compensation was also detected only at the GY and JR sites (Fig. 4). Thus, although the major factor governing site-specific temporal variation in *P. farinosa* is inconclusive, our results suggest that vital rate buffering and demographic compensation differ among sites, which likely contributes to the differential temporal λ variation among sites.

Site-specific demographic compensation could be due to the differential plastic responses of vital rates to the same environmental fluctuation, distinctive temporal environmental fluctuation, or both. Notably, a study testing the same *P. farinosa* populations used in this study showed that the plastic responses of growth and survival to temperature and soil nitrogen deposition are population-specific in a growth chamber environment^20^. The plasticity indices of growth for the GY and JR populations were greater than those for the other populations. Because demographic compensation is observed only at the GY and JR sites, it might depends on the degree of plasticity of the vital rates. Korean *P. farinose* populations exhibit higher genetic differentiation than other *Primula* species^25^, likely contributing to the differential plasticity of vital rates among sites.

Alternatively, micro-environmental conditions and their yearly fluctuations may differ among sites, inducing population-specific temporal demographic variation. Microclimatic conditions are a major factor affecting population demographics^38,39^. Unfortunately, we could not examine this hypothesis because of a lack of microclimatic information. The surrounding vegetation of *P. farinosa* populations could be another attribute of the distinctive demography. *P. farinosa* at the HL and CH sites occur on bare grassland with a few shrubs. By contrast, plants at the GY and JR sites occur with *Sanguisorba hakusanensis* and *Carex humilis*. Because of the low stature of *Primula* species, their vital rates are sensitive to the growth of surrounding vegetation^40,41^. The distinctive surrounding vegetation between testing sites might induce differential temporal demographic variations.

Effects of vital rates on the yearly λ variation differ between the GY and JR sites where vital rate buffering and demographic compensation are found. For instance, the Se growth and RA stasis buffer yearly λ variation at site JR, but at site GY, the SV survival and LV stasis buffer yearly λ variation (Fig. 3). Demographic compensation related to stasis reduced the variance in stochastic λ only at site JR (Fig. 5). These results further emphasize the need for studies at the population level for the conservation management of high-mountain species because the management of plants aims to increase the resilience of populations under fluctuating environments^8^. The results of manipulative studies, such as reciprocal transplants with environmental manipulation, can be combined with information from plant demography, which can reveal biological mechanisms causing site-specific effects of vital rates on temporal λ variation^42^.

In conclusion, *P. farinosa* exhibited site-specific yearly variation in the population growth rate, implying that high mountain populations might not decline simultaneously^29^. The contribution of vital rates to the yearly variation and patterns of demographic compensation also differed among the testing sites, which likely induced the site-specific temporal variation of λ. To conserve high mountain plant species, researchers should evaluate the temporal variation of populations in multiple locations separately.

## Methods

### Study sites and demographic survey

Detailed information on the study sites and the demographic survey is described in Jeong et al.^20^. We conducted demographic censuses from 2016 to 2021 at four sites: Cheonhwangsan (CH), Gayasan (GY), Hallasan (HL), and Jirisan (JR) (Supplementary Table S1). Permission for demographic survey was given by the Korea National Park Service. Experimental research on plants including the collection of plant material complied with relevant institutional and national guidelines and legislation. In June 2016, we established three 1 × 1 m^2^ plots at each site, and each plot contained 11–31 plants. A total of 225 plants were tagged. The number of individuals recorded over 6 years is presented in Supplementary Table S1. Regular censuses were conducted at the beginning (April and May) and end (August and September) of the growing season. All plants were marked in plots, and the following were recorded: the number of new seedlings, census to census survival, and morphological traits of plants, including rosette diameter, number of flowers, and fruits of all individuals in the plot.

### Matrix population model

We constructed stage-structured transition matrices for each site and year^27,28^. According to Lindborg and Ehrlén^26^, four life stages of *P. farinosa* were defined**:** seedling (Se), small vegetative plant (SV), large vegetative plant (LV), and reproductive adult (RA). The seedlings were newly observed individuals in the census at the beginning of the growing season. The minimum size (rosette diameter) of flowering plants was determined in each site and it was treated as a size threshold at which individuals could reproduce. Vegetative plants were classified as small or large based on the threshold size in each site, such that a vegetative plant smaller than the threshold size was the SV, and a vegetative plant larger than the threshold size was the LV. The seed stage was excluded in the matrix model because of absence of information, and it was combined with the seedling stage^26^. Combining the seed and seedling stages in the matrix model had a negligible effect on the population growth rate of other *P. farinosa* populations^26^. Fecundity was calculated as the number of seedlings in the following year divided by the number of RA. The elements (e_ij_) of the transition matrix correspond to the transition probability from stage j in year t to stage i in year t + 1, which was determined using monitoring data. Annual transition matrixes of each plant population are given in Supplementary Tables S2 and S3.

Vital rates underlying the elements of the transition matrices were calculated following Villellas et al.^12^ and Jongejans et al.^29^. The annual transition matrix model (A) using the vital rates is

1 $$\mathrm{A}= \left|\begin{array}{cc}\begin{array}{cc}\begin{array}{c}\begin{array}{c}\\ Se\end{array}\\ \begin{array}{c}SV\\ LV\\ F\end{array}\end{array}& \begin{array}{c}\begin{array}{c}Se\\ 0\end{array}\\ \begin{array}{c}{s}_{1}{g}_{21}\\ {s}_{1}{g}_{31}\\ {s}_{1}{g}_{41}\end{array}\end{array}\end{array}& \begin{array}{ccc}\begin{array}{c}\begin{array}{c}SV\\ 0\end{array}\\ \begin{array}{c}{s}_{2}{t}_{22}\\ {s}_{2}{g}_{32}\\ {s}_{2}{g}_{42}\end{array}\end{array}& \begin{array}{c}\begin{array}{c}LV\\ 0\end{array}\\ \begin{array}{c}{s}_{3}{r}_{23}\\ {s}_{3}{t}_{33}\\ {s}_{3}{g}_{43}\end{array}\end{array}& \begin{array}{c}\begin{array}{c}F\\ f\end{array}\\ \begin{array}{c}{s}_{4}{r}_{24}\\ {s}_{4}{r}_{34}\\ {s}_{4}{t}_{44}\end{array}\end{array}\end{array}\end{array}\right|,$$

where matrix elements are combinations of five vital rates: annual survival (*s*_*i*_) of plants in a life stage, the conditional probability of growth (*g*_*ij*_) to larger or flowering life stage given survival, the conditional probability of retrogression (*r*_*ij*_) to smaller or non-flowering life stage given survival, the conditional probability of stasis (*t*_*ij*_) in the same life stage given survival, and seedling emergence (*f*) as the same value of fecundity in the annual transition matrix^12,30^.

### Matrix analyses

All analyses were performed using R 4.1.2 (R Core Team, 2015). We calculated projected population growth rates using the package *popbio*^43^. Deterministic population growth rates (λ_d_) for each year and each site were calculated. Stochastic population growth rate (λ_s_) and its 95% confidence interval for each population were estimated by numerical simulation of 1000 time interval. At each time step, one of five annual matrices was randomly chosen with equal probabilities. The Mean matrix over years were built for each site, and elasticities of vital rates were calculated.

LTREs were conducted to compare the effects of site and year on λ. The two-way LTRE model was used to decompose the variation of λ into the effect of site m (*α*^*m*^), the effect of year n (*β*^*n*^), and their interaction (*αβ*^*mn*^)^14,27,29^**.** The model is

2 $${\lambda }^{mn} \cong {\lambda }^{..}+ {\alpha }^{m}+ {\beta }^{n}+ {\alpha \beta }^{mn},$$

where *λ*^*mn*^ is the deterministic population growth rate of site m and year n, and *λ*^*..*^ is the dominant eigenvalue of the mean of all the transition matrices. The main LTRE effects of site (α) and year (β) for each vital rate are defined by the difference between the vital rate of the matrix of interest and its corresponding vital rate of the appropriate reference matrix, multiplied by the sensitivity of λ calculated based on the means of the vital rate values of the reference matrix and the matrix of interest^30^. In this study, the mean matrix over sites and year was used as the reference matrix. The interaction term is defined by comparing individual matrices with the overall mean matrix and subtracting the associated main effects. The model is

3 $${\alpha }^{m.}={\lambda }^{m.}-{\lambda }^{..}\approx {\sum }_{i,j}{\sum }_{p}\left({v}_{p}^{m.}-{v}_{p}^{..}\right)\frac{\partial \lambda }{\partial {a}_{ij}}\frac{\partial {a}_{ij}}{\partial {v}_{p}}{|}_{\frac{1}{2}\left({A}^{m.}+ {A}^{..}\right)},$$

4 $${\beta }^{.n}={\lambda }^{.n}-{\lambda }^{..}\approx {\sum }_{i,j}{\sum }_{p}\left({v}_{p}^{.n}-{v}_{p}^{..}\right)\frac{\partial \lambda }{\partial {a}_{ij}}\frac{\partial {a}_{ij}}{\partial {v}_{p}}{|}_{\frac{1}{2}\left({A}^{.n}+ {A}^{..}\right)},$$

5 $${\alpha \beta }^{mn}={\lambda }^{mn}-{\lambda }^{..}- {\alpha }^{m.}-{\beta }^{.n}\approx {\sum }_{i,j}{\sum }_{p}\left({v}_{p}^{mn}-{v}_{p}^{..}\right)\frac{\partial \lambda }{\partial {a}_{ij}}\frac{\partial {a}_{ij}}{\partial {v}_{p}}{|}_{\frac{1}{2}\left({A}^{mn}+ {A}^{..}\right)}- {\alpha }^{m.}-{\beta }^{.n},$$

where *a*_*ij*_ is the matrix element in the ith row and jth column, and *v*_*p*_ is p^th^ vital rate.

To estimate the overall effects of year and site, the average of the individual year or site effects (net LTRE effect) and the average of absolute values of the individual year or site effects (gross LTRE effect) were calculated following Jongejans et al.^29^. In addition, the overall effects of year, site, and year × site interaction were decomposed into the contributions of vital rates because the magnitudes and directions of the impact of vital rates might differ^30^.

The effect of the site-by-year interaction on λ was larger than the main effects of site and year (see the “Results”), indicating that the observed λ of the individual matrix might differ from the expectation based on the site and year main effects. We performed an additional LTRE to analyze the year effect within the site, *α*(*β*)^*mn*^, on λ^m.^ for each site:

6 $${\alpha (\beta )}^{mn}\cong {\lambda }^{mn}-{\lambda }^{m.}\approx {\sum }_{i,j}{\sum }_{p}\left({v}_{p}^{mn}-{v}_{p}^{m.}\right)\frac{\partial \lambda }{\partial {a}_{ij}}\frac{\partial {a}_{ij}}{\partial {v}_{p}}{|}_{\frac{1}{2}\left({A}^{mn}+ {A}^{m.}\right)}.$$

The yearly variation in λ within each site was decomposed into the effects of vital rates in each life stage. The opposite concept of growth, stasis was calculated as the sum of stasis (*t*) and retrogression (*r*). We conducted two analyses to assess the demographic significance of vital rates in the temporal variation of λ. First, Spearman’s correlation coefficients were calculated between the LTRE effects of vital rates and the yearly deviation of λ for each site. A strong correlation indicates that the variation in these vital rates consistently contributes to the yearly variation in λ^30^. The statistical significance of the correlation coefficients was evaluated using a permutation test with 1000 simulations in the *wPerm* package^44^. Analyses were conducted separately for each life stage. To assess whether the correlation coefficients differed among sites, we conducted permutation analyses of covariance using the *permuco* package^45^. The model comprised the site, the LTRE effect of vital rate, and their interaction as fixed factors. A statistically significant site-by-vital rate interaction indicates that sites exhibit differential correlation coefficients.

Second, Spearman’s correlation coefficients were calculated between all pairs of LTRE contributions of vital rates in each life stage. Following Villellas et al.^12^, we examined correlations among LTRE contributions of vital rates instead of values of vital rates. Negative correlations indicate demographic compensation^12^. Statistical significance was evaluated using a permutation test in the *wPerm* package^44^. In addition, we created a null distribution of the number of significant negative correlations by using 10,000 random permutations of the contribution of vital rates and examined whether the number of significant negative correlations was higher than expected by chance, using the method of Villellas et al.^12^.

To assess the mitigation of temporal variation by negative correlations of vital rates, we created matrix lists by using 10,000 permutation samplings of focal vital rates with significant negative correlations. As in Villellas et al.^12^, we selected the focal vital rates based on one criterion—that the sum of the contribution of vital rates negatively correlated with the focal vital rate was greater than the sum of vital rates positively correlated with the focal vital rate—to preserve the relevant positive correlations as much as possible. The variance of stochastic λ (σ^2^_logλs_) was calculated from a randomized matrix^15^. If the σ^2^_logλs_ of the created matrix was greater than the observed σ^2^_logλs_, it would be interpreted as the buffered effects of negative correlation between vital rates on the temporal variation.

## Supplementary Information

Supplementary Information.

## Data Availability

All data generated or analysed during this study are included in this published article [and its supplementary information files]. The datasets used and/or analysed during the current study available from the corresponding author on reasonable request.
