# Supplementary material for: Site-specific temporal variation of population dynamics in subalpine endemic plant species
Source: Sci Rep. 2022 Nov 10;12:19207. doi: 10.1038/s41598-022-23903-5 (PMC9649610; doi:10.1038/s41598-022-23903-5)

Table S1. Locations of study sites and the number of *P. farinosa* individuals censused at each site.

| Sites | Location | Number of individuals censused | | | | |
| --- | --- | --- | --- | --- | --- | --- |
|  |  | 2016-2017 | 2017-2018 | 2018-2019 | 2019-2020 | 2020-2021 |
| Cheonhwangsan (CH) | 35.5577, 128.9731 | 48 | 50 | 52 | 55 | 59 |
| Gayasan (GY) | 35.8237, 128.1194 | 63 | 54 | 48 | 42 | 41 |
| Hallasan (HL) | 33.3632, 126.5139 | 45 | 54 | 48 | 34 | 34 |
| Jirisan (JR) | 35.3627, 127.8759 | 70 | 75 | 79 | 86 | 101 |

Table S2. Transition matrices of all years in CH and GY sites. Life stages are divided into seedling (Se), small vegetative plants (SV), large vegetative plants (LV), reproductive adults (RV), and fecundity (F).

| CH  (2016-2017) | Se | SV | LV | RA | GY  (2016-2017) | Se | SV | LV | RA |
| --- | --- | --- | --- | --- | --- | --- | --- | --- | --- |
| Se | 0.000 | 0.000 | 0.000 | 0.722 | Se | 0.000 | 0.000 | 0.000 | 0.456 |
| SV | 0.722 | 0.333 | 0.340 | 0.111 | SV | 0.167 | 0.194 | 0.059 | 0.000 |
| LV | 0.000 | 0.167 | 0.225 | 0.111 | LV | 0.667 | 0.583 | 0.634 | 0.267 |
| RA | 0.000 | 0.000 | 0.413 | 0.778 | RA | 0.000 | 0.000 | 0.080 | 0.650 |
| CH  (2017-2018) | Se | SV | LV | RA | GY  (2017-2018) | Se | SV | LV | RA |
| Se | 0.000 | 0.000 | 0.000 | 0.629 | Se | 0.000 | 0.000 | 0.000 | 0.367 |
| SV | 0.417 | 0.167 | 0.000 | 0.114 | SV | 0.667 | 0.000 | 0.037 | 0.000 |
| LV | 0.500 | 0.468 | 0.611 | 0.225 | LV | 0.000 | 0.667 | 0.568 | 0.389 |
| RA | 0.000 | 0.159 | 0.167 | 0.594 | RA | 0.000 | 0.000 | 0.233 | 0.417 |
| CH  (2018-2019) | Se | SV | LV | RA | GY  (2018-2019) | Se | SV | LV | RA |
| Se | 0.000 | 0.000 | 0.000 | 0.825 | Se | 0.000 | 0.000 | 0.000 | 0.540 |
| SV | 0.389 | 0.667 | 0.042 | 0.000 | SV | 0.000 | 0.500 | 0.000 | 0.000 |
| LV | 0.333 | 0.333 | 0.437 | 0.210 | LV | 0.583 | 0.278 | 0.410 | 0.301 |
| RA | 0.000 | 0.000 | 0.407 | 0.724 | RA | 0.000 | 0.000 | 0.269 | 0.651 |
| CH  (2019-2020) | Se | SV | LV | RA | GY  (2019-2020) | Se | SV | LV | RA |
| Se | 0.000 | 0.000 | 0.000 | 0.682 | Se | 0.000 | 0.000 | 0.000 | 0.429 |
| SV | 0.300 | 0.250 | 0.042 | 0.000 | SV | 0.000 | 0.000 | 0.000 | 0.000 |
| LV | 0.150 | 0.556 | 0.242 | 0.273 | LV | 0.083 | 0.000 | 0.150 | 0.143 |
| RA | 0.000 | 0.083 | 0.583 | 0.605 | RA | 0.444 | 0.333 | 0.633 | 0.738 |
| CH  (2020-2021) | Se | SV | LV | RA | GY  (2020-2021) | Se | SV | LV | RA |
| Se | 0.000 | 0.000 | 0.000 | 0.512 | Se | 0.000 | 0.000 | 0.000 | 0.437 |
| SV | 0.300 | 0.111 | 0.000 | 0.000 | SV | 0.111 | 0.333 | 0.000 | 0.000 |
| LV | 0.333 | 0.111 | 0.214 | 0.214 | LV | 0.000 | 0.167 | 0.083 | 0.242 |
| RA | 0.067 | 0.500 | 0.588 | 0.613 | RA | 0.583 | 0.000 | 0.500 | 0.590 |

Table S3. Table S2. Transition matrices of all years in JR and HL sites. Life stages are divided into seedling (Se), small vegetative plants (SV), large vegetative plants (LV), reproductive adults (RV), and fecundity (F).

| JR  (2016-2017) | Se | SV | LV | RA | HL  (2016-2017) | Se | SV | LV | RA |
| --- | --- | --- | --- | --- | --- | --- | --- | --- | --- |
| Se | 0.000 | 0.000 | 0.000 | 0.667 | Se | 0.000 | 0.000 | 0.000 | 1.111 |
| SV | 0.000 | 0.389 | 0.064 | 0.000 | SV | 0.000 | 0.222 | 0.225 | 0.233 |
| LV | 0.333 | 0.305 | 0.679 | 0.000 | LV | 0.367 | 0.528 | 0.297 | 0.167 |
| RA | 0.000 | 0.000 | 0.130 | 0.778 | RA | 0.067 | 0.250 | 0.393 | 0.600 |
| JR  (2017-2018) | Se | SV | LV | RA | HL  (2017-2018) | Se | SV | LV | RA |
| Se | 0.000 | 0.000 | 0.000 | 0.889 | Se | 0.000 | 0.000 | 0.000 | 0.331 |
| SV | 0.000 | 0.167 | 0.000 | 0.000 | SV | 0.383 | 0.333 | 0.089 | 0.083 |
| LV | 0.333 | 0.667 | 0.302 | 0.278 | LV | 0.250 | 0.111 | 0.421 | 0.271 |
| RA | 0.000 | 0.000 | 0.481 | 0.500 | RA | 0.217 | 0.361 | 0.282 | 0.509 |
| JR  (2018-2019) | Se | SV | LV | RA | HL  (2018-2019) | Se | SV | LV | RA |
| Se | 0.000 | 0.000 | 0.000 | 0.365 | Se | 0.000 | 0.000 | 0.000 | 0.294 |
| SV | 0.000 | 0.500 | 0.269 | 0.000 | SV | 0.444 | 0.083 | 0.095 | 0.000 |
| LV | 0.583 | 0.167 | 0.432 | 0.301 | LV | 0.222 | 0.333 | 0.538 | 0.224 |
| RA | 0.000 | 0.000 | 0.133 | 0.651 | RA | 0.111 | 0.528 | 0.312 | 0.693 |
| JR  (2019-2020) | Se | SV | LV | RA | HL  (2019-2020) | Se | SV | LV | RA |
| Se | 0.000 | 0.000 | 0.000 | 0.778 | Se | 0.000 | 0.000 | 0.000 | 0.655 |
| SV | 0.444 | 0.278 | 0.111 | 0.000 | SV | 0.444 | 0.083 | 0.095 | 0.061 |
| LV | 0.556 | 0.556 | 0.519 | 0.500 | LV | 0.222 | 0.333 | 0.538 | 0.194 |
| RA | 0.000 | 0.000 | 0.222 | 0.278 | RA | 0.111 | 0.528 | 0.312 | 0.667 |
| JR  (2020-2021) | Se | SV | LV | RA | HL  (2020-2021) | Se | SV | LV | RA |
| Se | 0.000 | 0.000 | 0.000 | 0.750 | Se | 0.000 | 0.000 | 0.000 | 0.670 |
| SV | 0.417 | 0.167 | 0.037 | 0.000 | SV | 0.425 | 0.111 | 0.000 | 0.000 |
| LV | 0.583 | 0.667 | 0.528 | 0.417 | LV | 0.350 | 0.500 | 0.421 | 0.231 |
| RA | 0.000 | 0.000 | 0.241 | 0.250 | RA | 0.000 | 0.389 | 0.510 | 0.702 |

Figure S1. Correlation diagrams between variation in the population growth rate (λ) and variation in the vital rate LTRE contribution to year effect. Separate analyses were conducted for each life stage. Spearman’s correlation coefficients were calculated for each site, and correlations without statistical significance (*P* > 0.05) by permutation test are represented by open symbols and dotted trend lines.


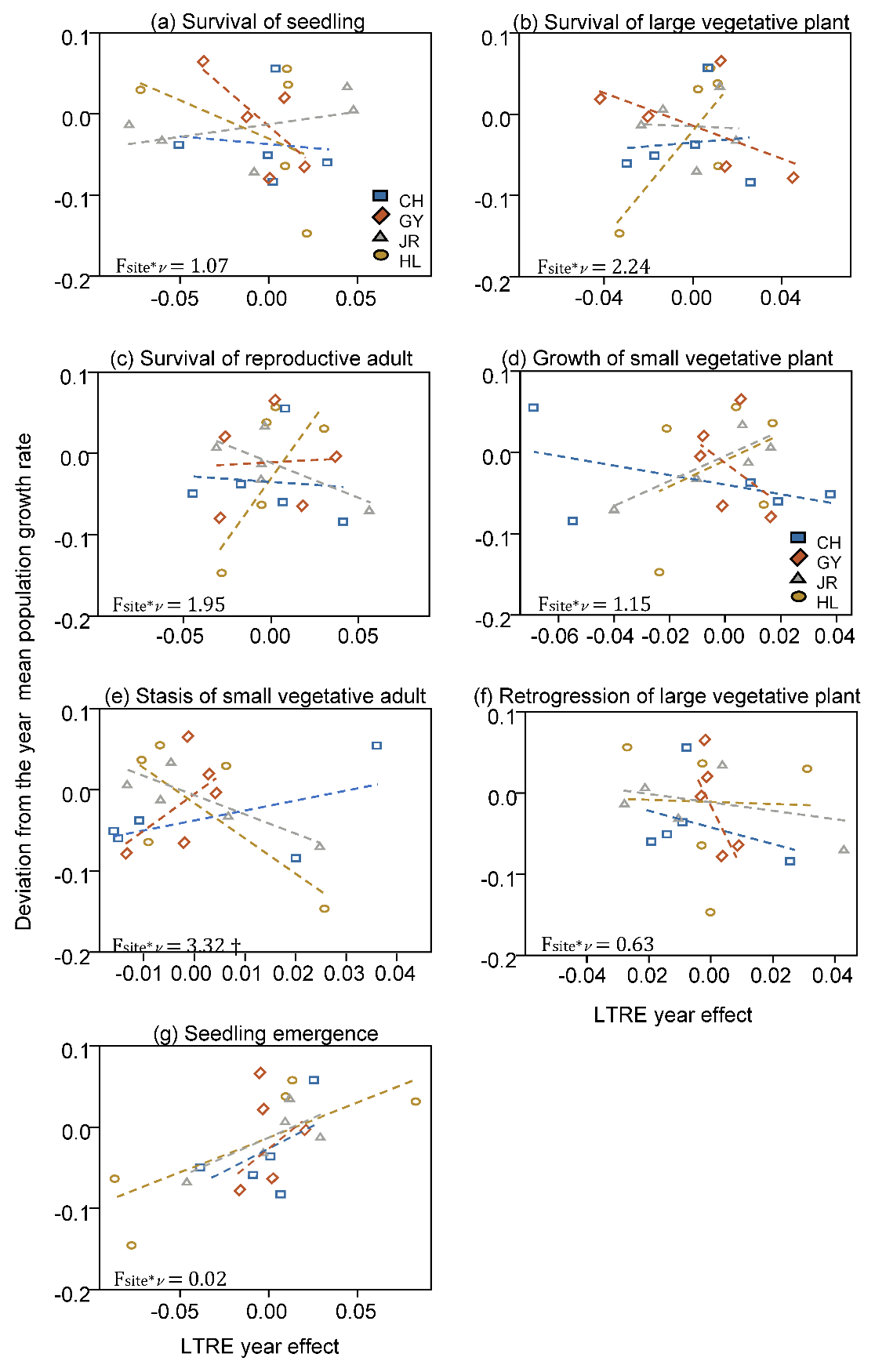

Supplement: Supplementary file 1 — Supplementary Information. [file 41598_2022_23903_MOESM1_ESM.docx]
